# Comprehensive pan-cancer analysis of STAT3 as a prognostic and immunological biomarker

**DOI:** 10.1038/s41598-023-31226-2

**Published:** 2023-03-28

**Authors:** Zhibo He, Biao Song, Manling Zhu, Jun Liu

**Affiliations:** 1 The School of Foreign Languages, Jiujiang University, Jiujiang, China; 2 Medical School, Jiujiang University, Jiujiang, China; 3 Laboratory of Precision Preventive Medicine, Jiujiang University, Jiujiang, China

**Keywords:** Cancer, Computational biology and bioinformatics

## Abstract

Numerous studies have indicated that STAT3 plays a key role in promoting oncogenesis and it is considered a potential therapeutic target for cancer treatment; however, there are no reports on STAT3 using pan-cancer analysis. Therefore, it is important to investigate the role of STAT3 in different types of tumors using pan-cancer analysis. In the present study, we used multiple databases to comprehensively analyze the relationship between STAT3 expression and prognosis, different stages of patients with cancer, investigate the clinical value of STAT3 in predicting prognosis, and the relationship between STAT3 genetic alteration and prognosis, drug sensitivity, and STAT3 expression, to determine whether STAT3 participates in tumor immunity, to provide a rationale for STAT3 as a treatment target for a broad-spectrum malignancies. Our results indicate that STAT3 can serve as a prognostic, sensitivity prediction biomarker and a target for immunotherapy, which has been of great value for pan-cancer treatment. Overall, we found that STAT3 significantly predicted cancer prognosis, drug resistance, and immunotherapy, providing a rationale for further experimental studies.

## Introduction

Currently, cancer is the first or second leading cause of death and is one of the leading disease-related causes of death worldwide. With the aging population, the morbidity and incidence rates of malignant tumors are increasing every year^1^. Given the current dire global cancer situation, immunotherapy is on the rise. On one hand, immunotherapy can increase the ability of the immune system to identify and eliminate cells with tumor antigens, enhancing immune-mediated anti-tumor immune responses; on the other hand, it can inhibit tumor-induced immune suppression^2^. Cancer immunotherapy has achieved dramatic results in patients with cancer; therefore, it is considered the third revolution in cancer treatment^3^. Thus, it is necessary to identify new biomarkers to prevent and treat cancer. However, statistical power and poor reproducibility limit the findings of many studies^4^. With the rapid development of tumor genomic technologies, cancer research has entered pan-cancer analysis. Pan-cancer analysis refers to the study of tumors of a variety of cancer types using genomics technology, finding common features across diverse cancer types, understanding tumor progression, and providing a new and promising therapeutic avenue for the treatment of a broad spectrum of cancers^5^.

Signal transducer and activator of transcription 3 (STAT3) is a member of the STAT protein family that acts as a transcriptional activator. STAT3 can be activated by Janus kinases (JAKs), epidermal growth factor receptor (EGFR), and interleukin 6 (IL6), which are involved in a number of physiological and pathological processes, including cell survival, proliferation, transformation, apoptosis, as cellular immunity^6^. In recent years, studies have shown that aberrant STAT3 phosphorylation has been implicated in malignant transformation of cells, mediating tumor initiation and progression, and is therefore considered a proto-oncogene^7^. It has been shown that aberrant expression of STAT3 has tumor-promoting effects in many cancers, such as squamous cell carcinoma^8^, thyroid cancer^9^, and non-small cell lung cancer ^10^, which is considered a significant hub in tumorigenesis. Similarly, in the tumor immune response, STAT3 phosphorylation can promote evasion of immune surveillance^11^, and inhibition of STAT3 phosphorylation can suppress evasion of immune surveillance^12^. Numerous studies have indicated that STAT3 plays a key role in promoting oncogenesis, and it is considered a potential therapeutic target for cancer treatment; however, there are no reports on STAT3 using pan-cancer analysis. Therefore, it is important to investigate the role of STAT3 in different types of tumors using pan-cancer analysis.

In the present study, we comprehensively used multiple databases to analyze the relationship between STAT3 expression and prognosis, different stages of patients with pan-cancer, investigate the clinical value of STAT3 in predicting prognosis, and the relationship between STAT3 genetic alteration and prognosis, drug sensitivity, and STAT3 expression, to determine whether STAT3 participates in tumor immunity, to provide a rationale for STAT3 as a treatment for broad-spectrum malignancy.

## Materials and methods

### Pan-cancer expression analysis

The Sangerbox databases contain the expression of STAT3 genes in human cancer and normal tissues in TCGA and GTEx (genotype-tissue expression). After removing instances with fewer than three samples, the expression of STAT3 in normal vs. cancer tissues was visualized using the Sangerbox database (http://vip.sangerbox.com/). The UALCAN portal (http://ualcan.path.uab.edu/) was used to conduct the protein expression analysis of the CPTAC (Clinical Proteomic Tumor Analysis Consortium) dataset. Then, in the GEPIA2 database (http://gepia2.cancer-pku.cn/#analysis), STAT3 was added as input and cancers in pan-cancer were selected; STAT3 expression information in different stage of patients with cancer were obtained in “Expression DIY-StagePlot.”

### Survival prognosis analysis

In the GEPIA2 database (http://gepia2.cancer-pku.cn/#analysis), STAT3 was added as input, cancers in pan-cancer were selected, and a cut-off value of 50% was selected as the dividing threshold. The relationship between STAT3 expression and “Overall Survival” and “Recurrence-free survival” was collected in “Survival Analysis.”

### Genetic alteration analysis

Information on *STAT3* genetic alteration was obtained from the cBioPortal database (https://www.cbioportal.org/). In the cBioPortal database (https://www.cbioportal.org/), we entered “STAT3” in the “TCGA Pan-Cancer Atlas Studies” section for queries of the genetic alteration characteristics across all TCGA tumors. In the “Mutations” module, the mutated site information was displayed. Moreover, we also applied the “Comparison” to obtain data on the overall, disease-free, progression-free, and disease-free survival differences for cancer cases. The corresponding Kaplan–Meier plots with log-rank *P* values were generated for significantly different cancers with or without STAT3 genetic alterations.

### Protein phosphorylation and DNA methylation analysis

Using the UALCAN database (http://ualcan.path.uab.edu/), we investigated the total degree of proteomic expression and phospho-protein expression of STAT3 in cancer and normal tissues. STAT3 was added as an input and “TCGA” was selected, the information of STAT3 methylation in normal vs. cancer tissues was collected in “Methylation.” Then, “CPTAP” was selected to collect STAT3 protein phosphorylation data in normal and cancer tissues.

### Immune infiltration analysis

In the TIMER database (http://timer.cistrome.org/), we selected choosing "GENE,” STAT3 added as input in Gene Expression, and choosing “Cancer-associated fibroblast” in Immune Infiltrates, “Purity Adjustment” was used to obtain the relationship between STAT3 expression and cancer-associated fibroblasts. Relationship between STAT3 expression and cancer-associated immune infiltration score was obtained in Sangerbox database (http://vip.sangerbox.com/), and the results were considered to be significant when *P*-value was < 0.05, and the absolute value of R was > 0.20^13^.

### STAT3‑related genes and protein enrichment analysis

In STRING database (https://cn.string-db.org/), STAT3 was used as input in the “multiple proteins” box with “Homo sapiens” selected as the species. In addition, the following parameters were set: meaning of network edges: “evidence,” active interaction sources: “Experiments,” minimum required interaction score: “low confidence 0.15” and max number of interactors to show: “no more than 50 interactors” ^14^. Finally, the results were visualized using Cytoscape 3.9.0. After that, in the “Similar Gene Detection” module of GEPIA2, we obtained the top 100 CBX3-correlated target genes. We then acquired five corresponding dot plots marked with the *P* value and correlation coefficient (R). Moreover, based on the “Gene_Corr” module in the “Exploration” of TIMER2.0, we obtained heatmap data of the five genes in TCGA cancers. We combined these two sets of data to perform Gene Ontology (GO) enrichment analysis and Kyoto Encyclopedia of Genes and Genomes (KEGG)^15^ in the DAVID database (https://david.ncifcrf.gov/). The DAVID database was entered into the gene list as a screening tool. “OFFICIAL-GENE-SYMBOL” was selected, “Homo sapiens” was set as the species selection, and *P* < 0.01 and FDR < 0.01 were used as the screening standards to investigate the signaling pathways and biological properties. The data were analyzed using visualization approaches available on the Weishengxin online platform (http://www.bioinformatics.com.cn/).

### Drug sensitivity analysis

The relationship between drug sensitivity and expression of STAT3 was obtained from GSCA database (http://bioinfo.life.hust.edu.cn/GSCA/#/drug), STAT3 was used as keyword input, selected “GDSC drug sensitivity and expression correlation”, then, the relationship between drug sensitivity and expression of STAT3 was obtained.

## Results

### STAT3 has differential expression between patients with cancer and controls

**Figure 1:**
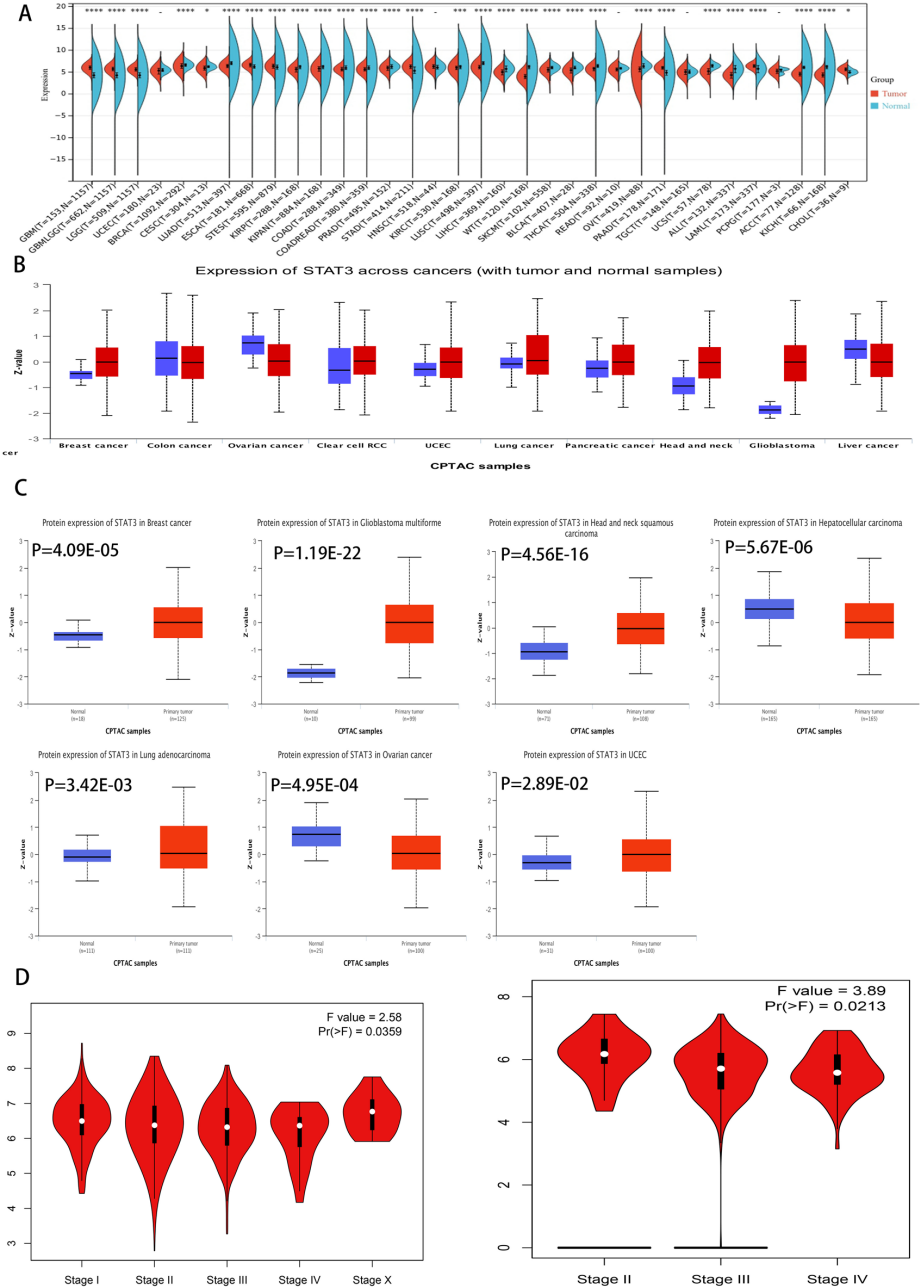
Analysis of differential expression of STAT3 in pan-cancer tissues and normal tissues. (**A**) STAT3 expression in normal and tumor tissues in TCGA and GTEx, blue represents normal, red represents tumor (**B**) STAT3 protein expression in normal and tumor tissues (UALCAN database), blue represents normal, red represents tumor (**C**) STAT3 protein expression in normal and tumor tissues box plots, blue represents normal, red represents tumor (*P* < 0.05) (**D**) The results of differential expression in normal tissues and breast cancer tissues (left, *P* < 0.05), the results of differential expression in normal tissues and Ovarian cancer tissues (right, *P* < 0.05).

The full names of the abbreviations and detailed information on cancer are summarized in Supplementary Table S1. The results showed that STAT3 mRNA expression differed between patients with cancer and controls in the Sangerbox database. (Fig. 1A). After removing instances with fewer than three samples, the mRNA expression profiles of the 34 cancer samples were obtained. We found significantly increased mRNA expression of STAT3 in nine tumor tissues, including glioblastoma multiforme (GBM, *P* = 1.1 e−71), glioma (GBMLGG *P* = 2.9 e−195), brain lower-grade glioma (LGG *P* = 2.1 e−158), esophageal carcinoma (ESCA *P* = 3.2 e−9), stomach and esophageal carcinoma (STES *P* = 1.3 e−8), stomach adenocarcinoma(STAD *P* = 2.5 e−22), pancreatic adenocarcinoma (PAAD *P* = 1.1 e−29),acute myeloid leukemia (LAML *P* = 1.2 e−13), and cholangiocarcinoma (CHOL *P* = 0.03). We found significantly decreased mRNA expression of STAT3 in nine tumor tissues, including breast invasive carcinoma (BRCA *P* = 4.2 e−8), cervical squamous cell carcinoma and endocervical adenocarcinoma (CESC *P* = 0.04), lung adenocarcinoma (LUAD *P* = 1.8 e−56), kidney renal papillary cell carcinoma (KIRP *P* = 9.3 e−14), pan-kidney (KIPAN* P* = 1.3 e−10), and colon adenocarcinoma (COAD *P* = 1.2 e−12). The ULACAN database results (Fig. 1B) showed that in the vast majority of these cancers, STAT3 proteins were overexpressed (Fig. 1C), such as in breast invasive carcinoma (BRCA, *P* = 4.09E−05), uterine corpus endometrial carcinoma (UCEC *P* = 2.89E−02), lung adenocarcinoma (LUAD *P* = 3.42E−03), head and neck squamous carcinoma (HNSC *P* = 4.56E−16), pancreatic adenocarcinoma (PAAD *P* = 2.77E−03), and glioblastoma multiforme (GBM *P* = 1.19E−22). Ovarian cancer (OV* P* = 4.95E−04) and liver hepatocellular carcinoma (LIHC *P* = 5.67E−06) had lower protein expression levels than normal tissues. The GEPIA2 database showed that the expression levels of STAT3 were different among the different breast invasive carcinoma (BRCA, *P* = 0.0359) and ovarian serous cystadenocarcinoma (OV, *P* = 0.0213) stages (Fig. 1D).

### STAT3 expression has been associated with prognosis

**Figure 2:**
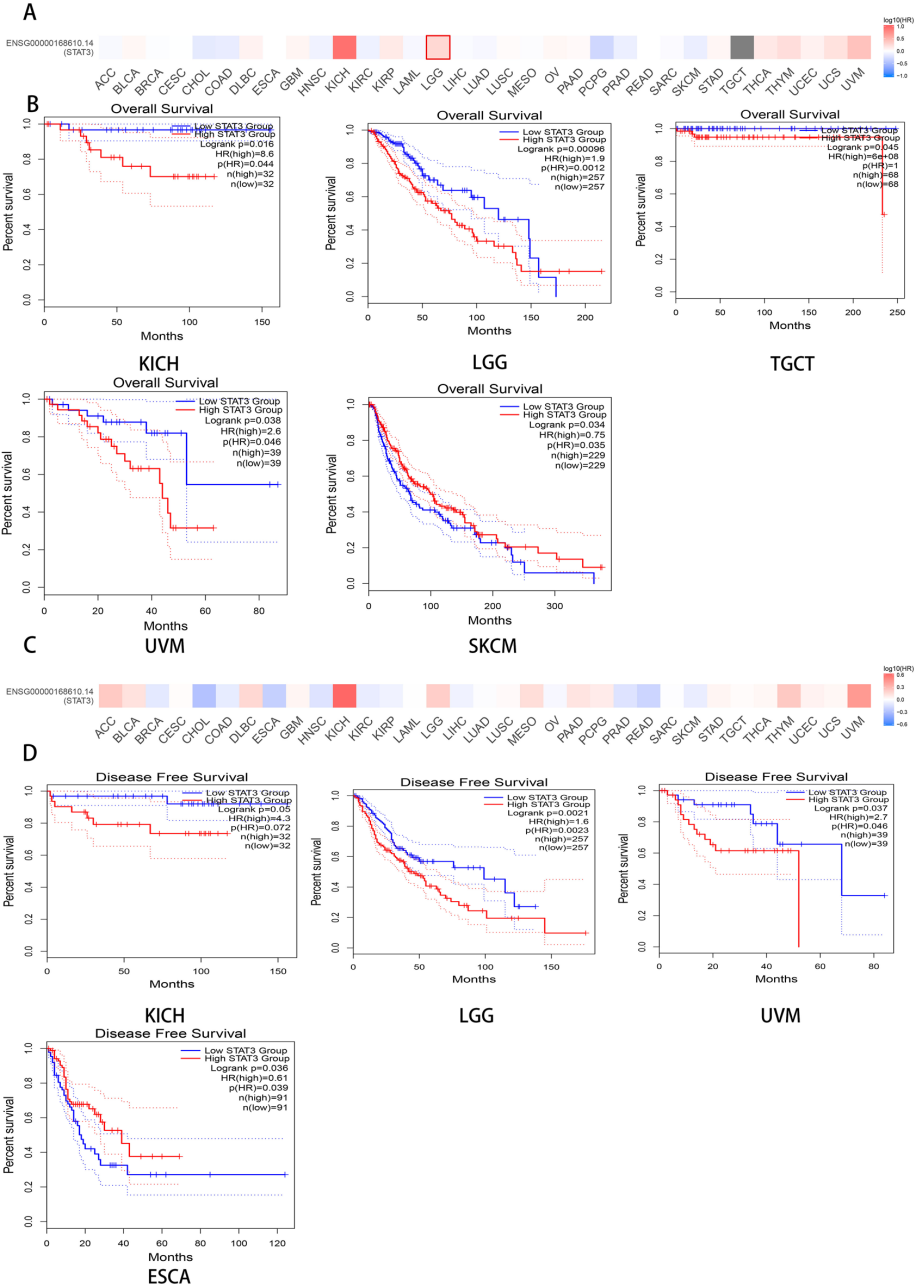
The result of survival prognosis analysis, (**A**) the relationship between different STAT3 expression levels and overall survival heat map. (**B**) the relationship between different STAT3 expression levels and overall survival K-M plots (*P* < 0.05), (**C**) the relationship between different STAT3 expression levels and Recurrence free survival heat map (**D**) the relationship between different STAT3 expression levels and Recurrence free survival (*P* < 0.05).

In OS, the GEIPA2 database showed (Fig. 2A, B) that in the vast majority of cases, patients with kidney chromophobe (KICH *P* = 0.016 HR = 8.6), brain lower grade glioma (LGG *P* = 0.00096 HR = 1.9), testicular germ cell tumors (TGCT *P* = 0.045, HR = 6e + 08), and uveal melanoma (UVM *P* = 0.038 HR = 2.6) with high levels of STAT3 expression had a significantly worse median survival time than those with low expression. However, in the vast majority of cases, patients with skin cutaneous melanoma (SKCM; *P* = 0.034, HR = 0.75) with high levels of STAT3 expression had a significantly better median survival time than those with low expression. In DFS (Fig. 2C, D), patients with kidney chromophobe (KICH, *P* = 0.05, HR = 4.3), brain lower grade glioma (LGG, *P* = 0.0021 HR = 1.6), and uveal melanoma (UVM, *P* = 0.037 HR = 2.7) with high levels of STAT3 expression had a significantly worse median survival time than those with low expression in the vast majority of cases. However, in the vast majority of cases, patients with esophageal carcinoma (ESCA *P* = 0.0036 HR = 0.61) with high levels of STAT3 expression had a significantly better median survival time than those with low expression.

### STAT3 gene is altered in multiple cancers

**Figure 3:**
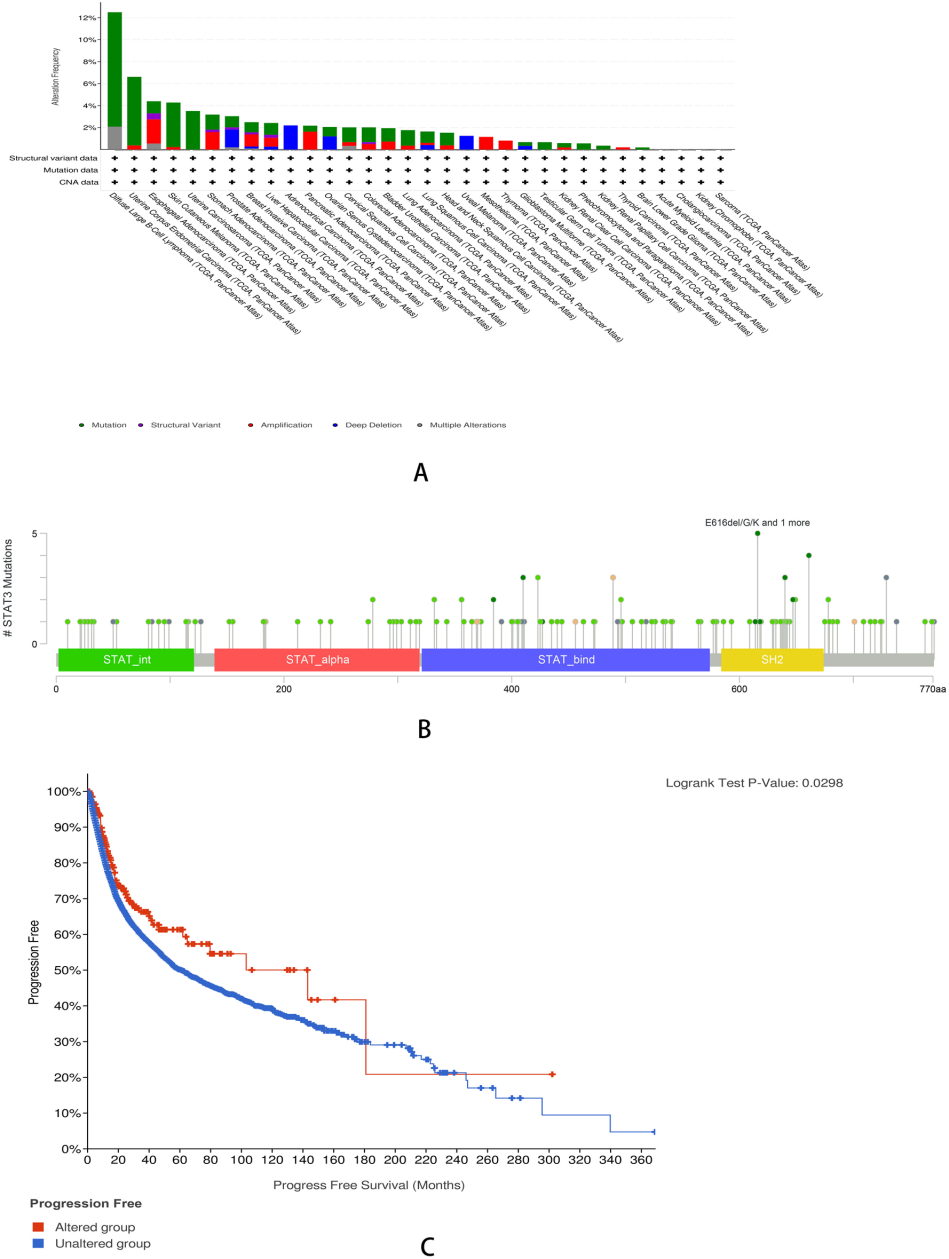
Mutation feature of STAT3 in different tumors. (**A**) the result of alteration analysis, green represents mutation, purple represents structure variant, red represents amplification, blue represents mutation Deep deletion, gray represents Alterations (**B**) STAT3 mutation site. (**C**) potential correlation between mutation status and progression-free survival of STAT3(*P* < 0.05).

The cBioPortal database results showed that STAT3 gene is altered in multiple cancers, and “Mutation” had the highest percentage. Diffuse large B-cell lymphoma had the highest alteration frequency, followed by uterine corpus endometrial carcinoma, which had the highest frequency. Then, we have noted that in adrenocortical carcinoma and uveal melanoma, the STAT3 gene alteration all were “Deep Deletion.” In mesothelioma and thymoma, all were “Amplification.” (Fig. 3A). The STAT3 gene mutations are shown in Fig. 3B. In addition, the results showed that in the vast majority of cases, the altered group had a significantly better median survival time than the unaltered group (*P* < 0.05) (Fig. 3C).

### STAT3 DNA methylation and protein phosphorylation differ in malignant versus normal tissue

**Figure 4:**
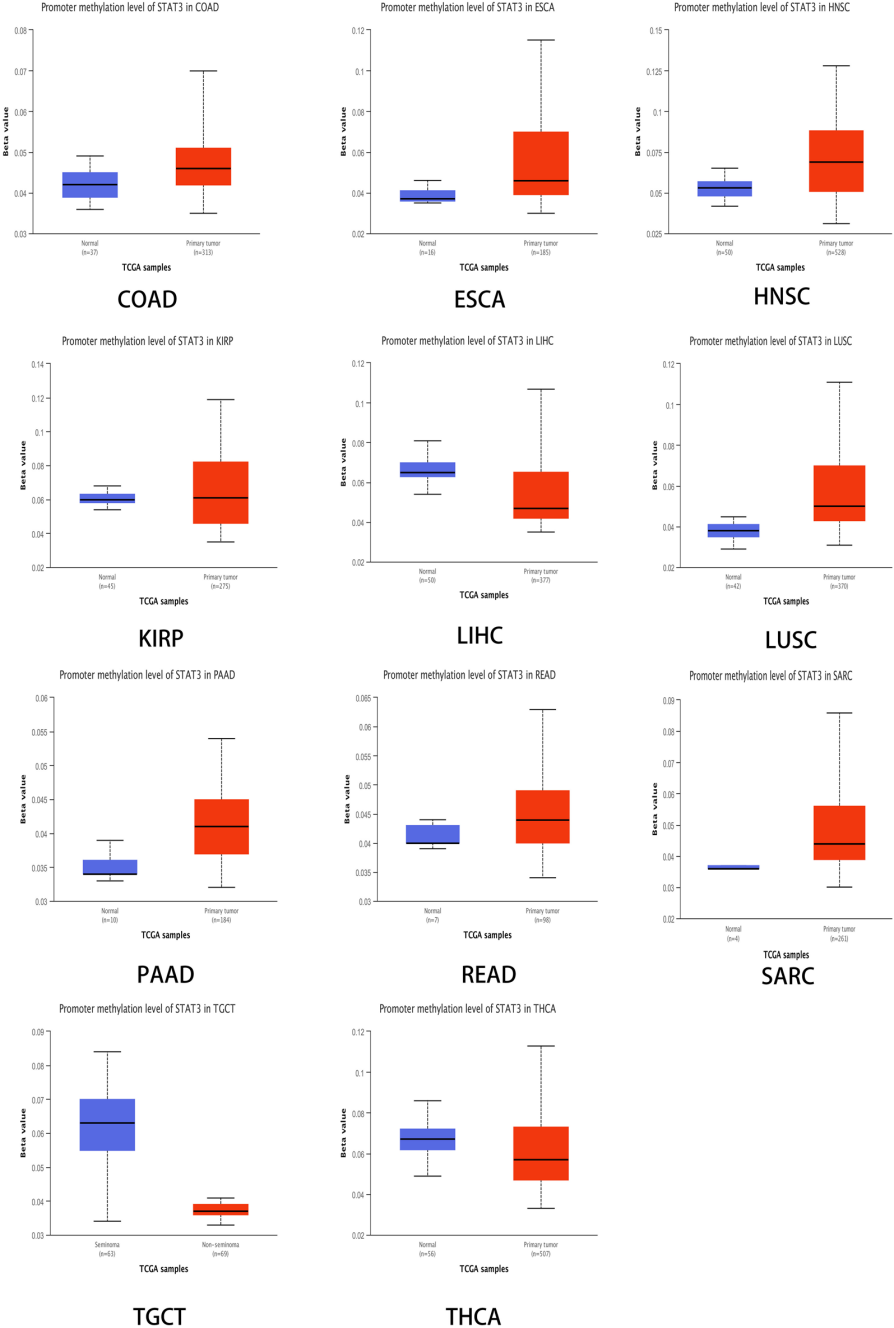
STAT3 DNA methylation level in normal and tumor tissues box plot, blue represents normal, red represents tumor (*P* < 0.05).

**Figure 5:**
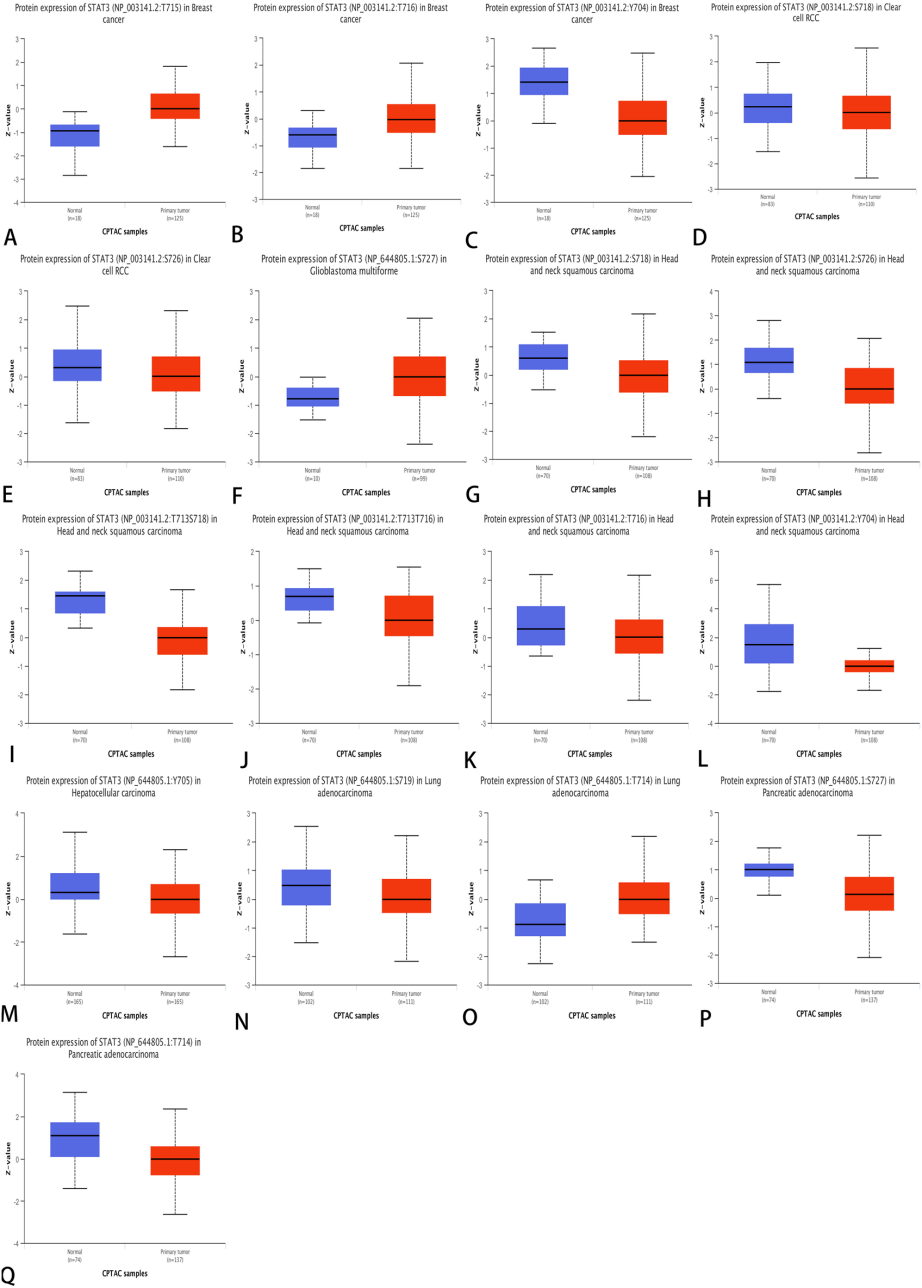
STAT3 Tumor-associated protein phosphorylation of STAT3. Box plot representation of STAT3 phosphorylation levels at different amino acid, blue represents normal, red represents tumor (*P* < 0.05). (**A**)–(**C**): Breast invasive carcinoma, (**D**)–(**E**): Kidney renal clear cell carcinoma, (**F**): Glioblastoma multiforme, (**G**)–(**L**): Head and Neck squamous cell carcinoma, (**M**): Liver hepatocellular carcinoma, (**N**)–(**P**): Lung adenocarcinoma (**Q**): Pancreatic adenocarcinoma.

We obtained results from the UALCAN database (Fig. 4). Owing to the limited number of tumors analyzed using our tools, only specific tumors were analyzed. We observed a significant increase in DNA methylation levels in colon adenocarcinoma (COAD), esophageal carcinoma (ESCA), head and neck squamous cell carcinoma (HNSC), kidney renal papillary cell carcinoma (KIRP), lung squamous cell carcinoma (LUSC), pancreatic adenocarcinoma (PAAD), rectal adenocarcinoma (READ), and sarcoma (SARC) compared to normal tissues (*P* < 0.05). In liver hepatocellular carcinoma (LIHC), testicular germ cell tumors (TGCT), and thyroid carcinoma (THCA), we observed a significantly lower DNA methylation level than in cancer tissues (*P* < 0.05). Subsequently, we compared the different phosphorylation features of STAT3 in normal and tumor tissues using the CPTAC dataset. STAT3 phosphorylation data for various tumor types are shown in Fig. 5 (Fig. 5A–Q), and the levels were significantly different in various tumors. STAT3 phosphorylation at sites T715 and T716 was higher, but it was lower at Y704 than those in normal tissues in breast invasive carcinoma (BRCA). In clear cell renal cell carcinoma (KIRC), the levels of phosphorylation at sites S718 and S726 were lower than those in normal tissues. In glioblastoma multiforme (GBM), phosphorylation at site S727 was higher, but in head and neck squamous carcinoma (HNSC), phosphorylation at sites Y704, S718, T713S718, S726, T713T716, and T716 were lower than those in normal tissues, as well as phosphorylation at site Y705 in liver hepatocellular carcinoma (LIHC) and phosphorylation at site S719 in lung adenocarcinoma (LUAD). However, the level of phosphorylation at site T714 was higher than that in normal tissues. In pancreatic adenocarcinoma (PAAD), the levels of phosphorylation at sites T714 and S727 were lower than those in normal tissues. All *P* -values were less than 0.05.

### STAT3 expression level is associated with immune infiltration in multiple cancer types

**Figure 6:**
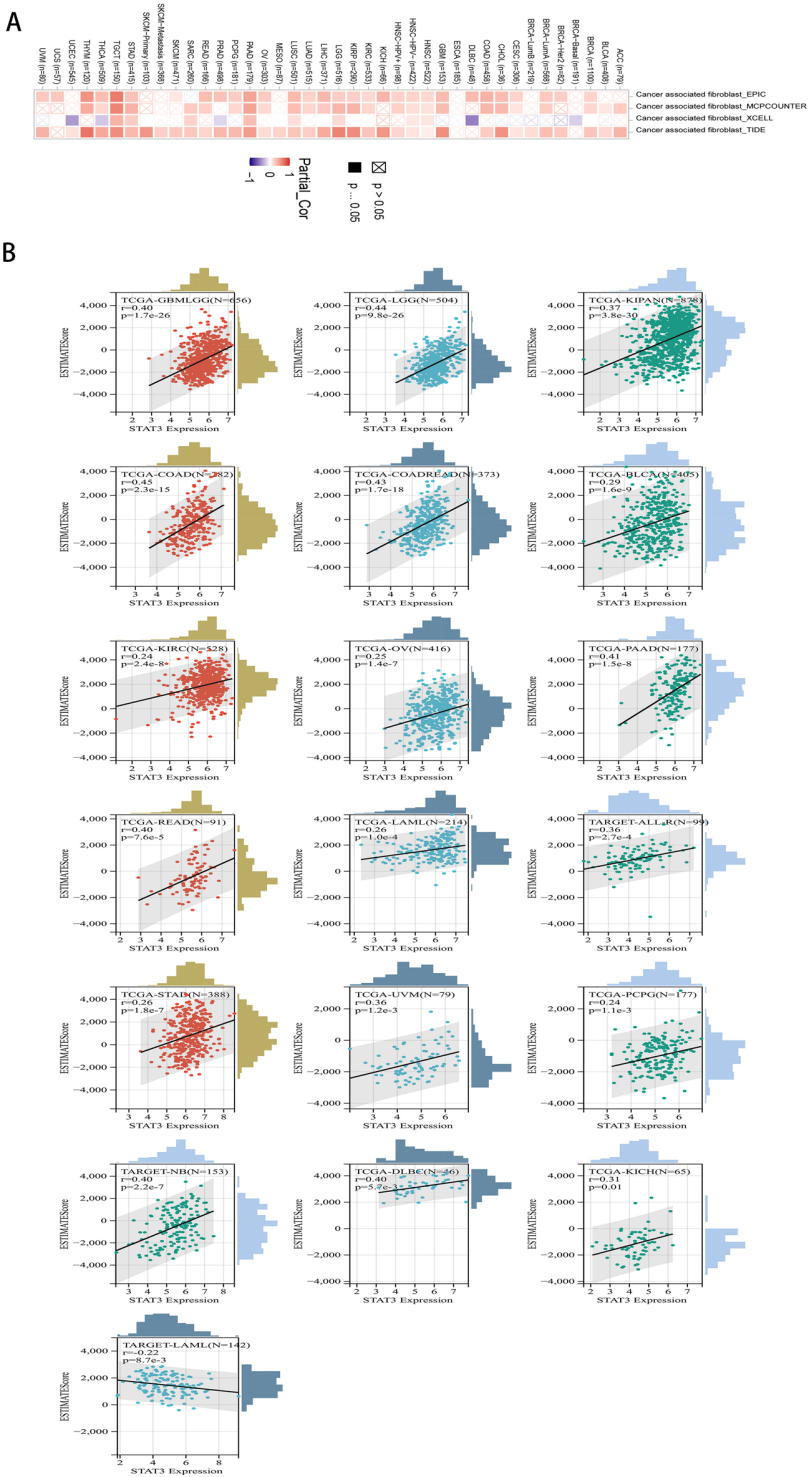
Pan-cancer association analysis of STAT3 expression and tumor immune infiltration. (**A**) The relationship between expression of STAT3 and cancer-associated fibroblasts heat map. (**B**) The relationship between expression of STAT3 and tumor immune infiltration scatter plot.

In the TIMER database, the same results across the algorithms (Fig. 6A) showed that the expression of STAT3 and cancer-associated fibroblasts showed an inverse correlation in ESCA (*P* < 0.05). However, in HPV-negative HNSCC (HNSC-HPV-), brain lower grade glioma (LGG), lung squamous cell carcinoma (LUSC), pancreatic adenocarcinoma (PAAD), stomach adenocarcinoma (STAD), testicular germ cell tumors (TGCT), and the expression of STAT3 and cancer-associated fibroblasts showed a positive correlation (*P* < 0.05). Sangerbox database results in Fig. 6B show that STAT3 expression status was significantly correlated with immune infiltration score in 27 cancers, 25 positive significant correlations, such as glioma (GBMLGG R = 0.40, *P* = 1.7 e−26), brain lower grade glioma (LGG R = 0.44, *P* = 9.8 e−26),, pan-kidney cohort (KIPAN R = 0.37, *P* = 3.8 e−30), colon adenocarcinoma (COAD R = 0.45, *P* = 2.3 e−15), colon adenocarcinoma/rectum adenocarcinoma esophageal carcinoma (COADREAD R = 0.43, *P* = 1.7 e−18), stomach adenocarcinoma (STAD R = 0.26, *P* = 1.8 e−7), kidney renal clear cell carcinoma (KIRC R = 0.24, *P* = 2.4 e−8), bladder urothelial carcinoma (BLCA R = 0.29, *P* = 1.6 e−9), neuroblastoma (NB R = 0.40, *P* = 2.2 e−7), rectum adenocarcinoma (READ R = 0.40, *P* = 7.6 e−5), ovarian serous cystadenocarcinoma (OV R = 0.25, *P* = 1.4 e−7), uveal melanoma (UVM R = 0.36, *P* = 1.2 e−3), pancreatic adenocarcinoma (PAAD R = 0.41, *P* = 1.5 e− e−8), acute myeloid leukemia (LAML R = 0.26, *P* = 1.0 e−4), pheochromocytoma and paraganglioma (PCPG R = 0.24, *P* = 1.1 e−3), acute lymphoblastic leukemia, recurrent blood-derived cancer, bone marrow (ALL-R R = 0.36, *P* = 2.7 e−4), lymphoid neoplasm diffuse large B-cell lymphoma (DLBC R = 0.40, *P* = 5.7 e−3), kidney chromophobe (KICH R = 0.31, *P* = 0.01), and a negative significant correlation such as TARGET-acute myeloid leukemia (LAML R = 0.22, *P* = 8.7 e−3).

### STAT3‑related genes and protein enrichment analysis

**Figure 7:**
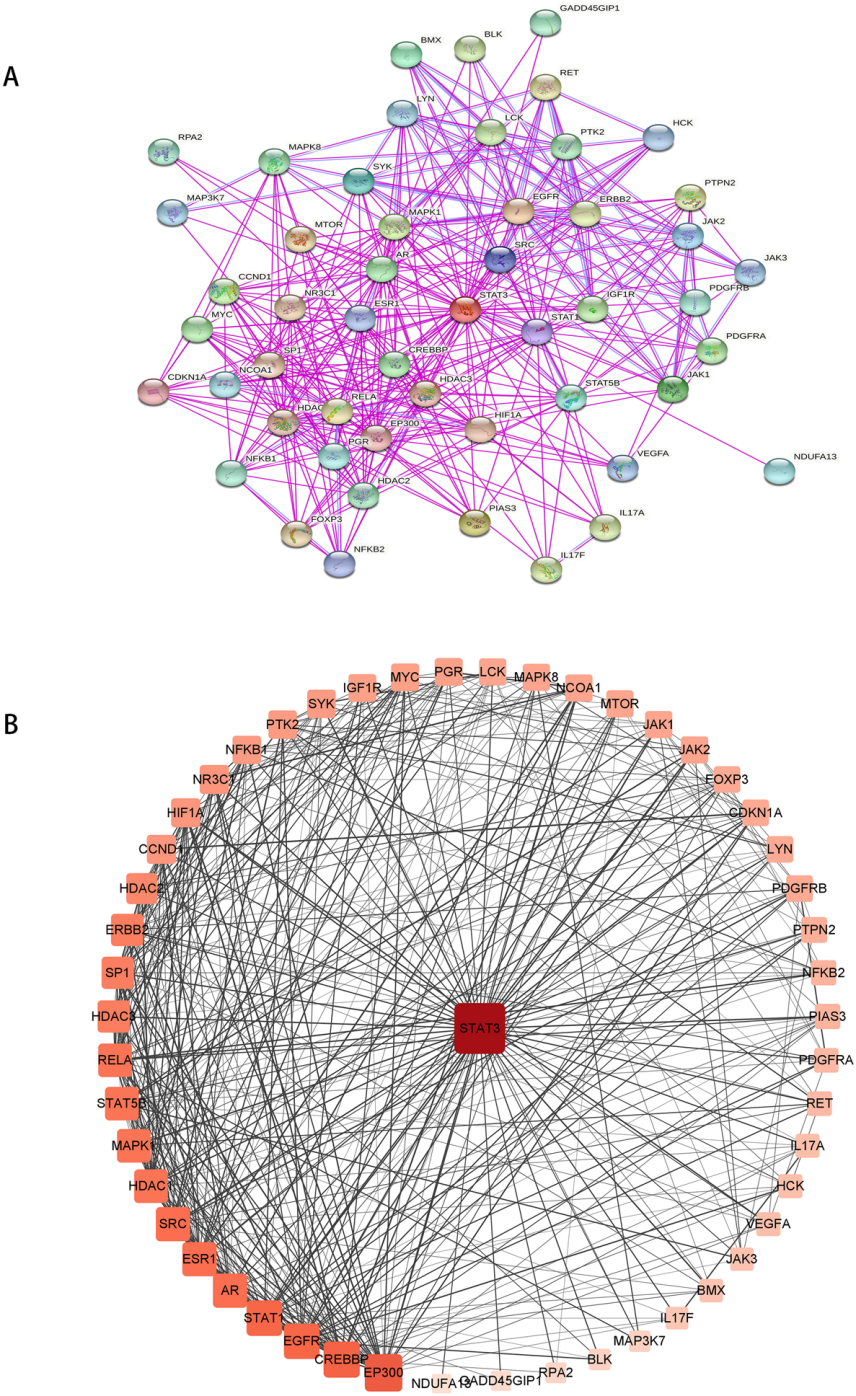
(**A**) protein–protein interaction networks of STAT3 in STRING. (**B**) after analysis in Cytoscape 3.9.0. The darker the color, the greater the degree, and the large area, the greater the degree.

**Figure 8:**
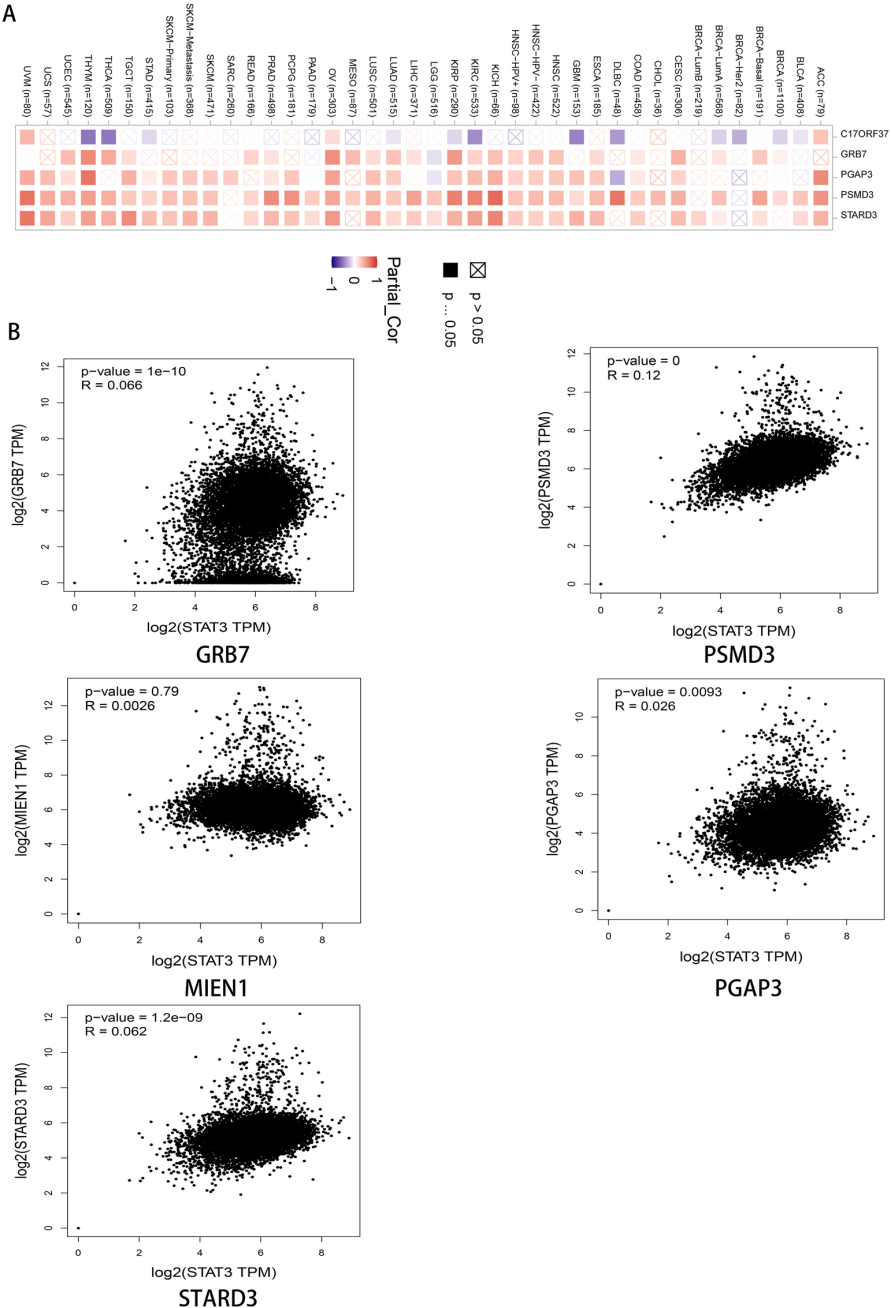
the expression correlation between STAT3 and selected targeting genes. (**A**) the expression correlation between STAT3 and selected targeting genes heat map, (**B**) the expression correlation between STAT3 and selected targeting genes scatter plot.

**Figure 9:**
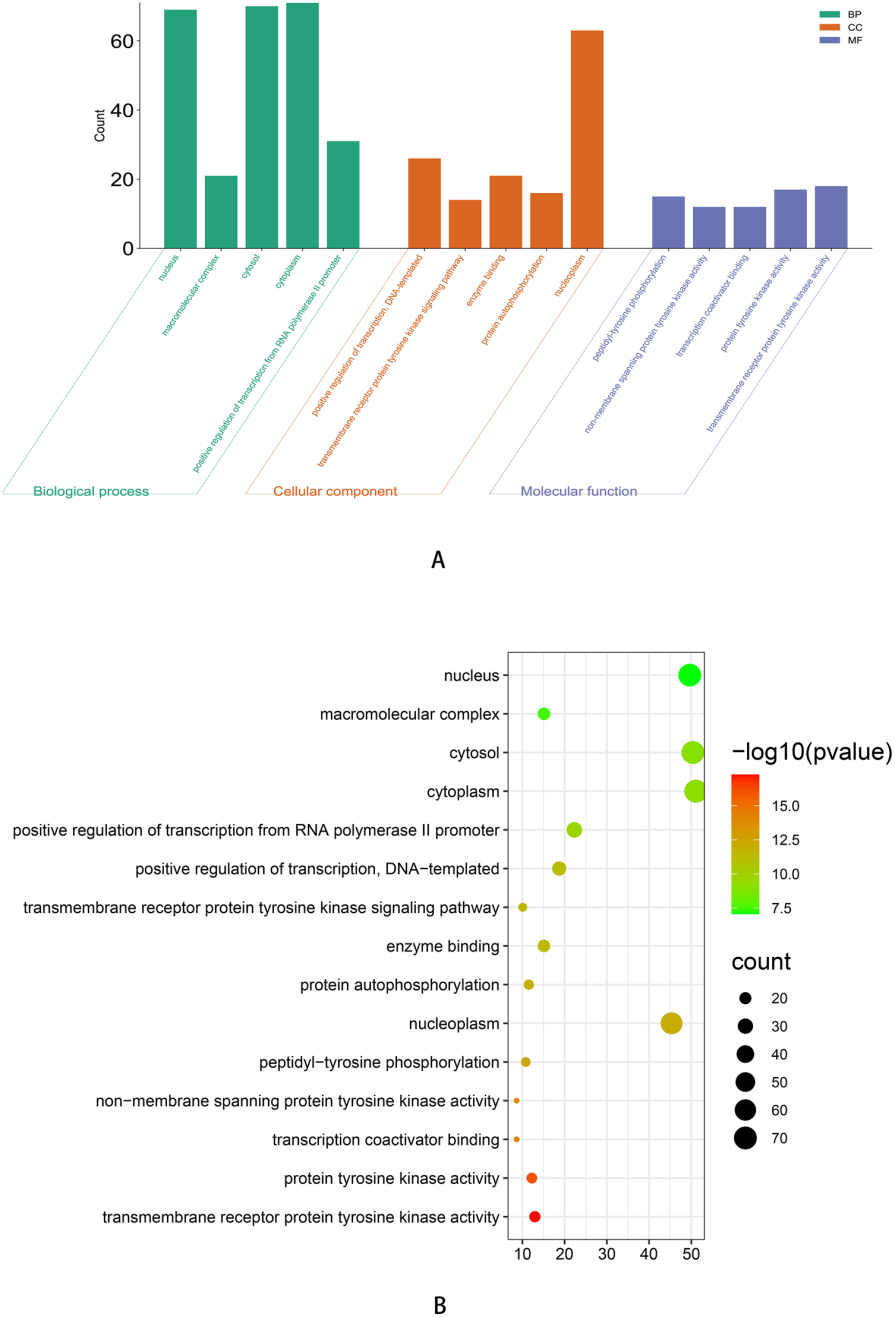
GO analysis of STAT3 related genes. GO: gene ontology, (**A**) Enrichment histogram, BP: biological processes; CC: cellular components; MF: molecular functions. (**B**) Enrichment dot bubble diagram, (the larger the circular dots, the more genes contained, the darker the color, the smaller *P* value).

**Figure 10:**
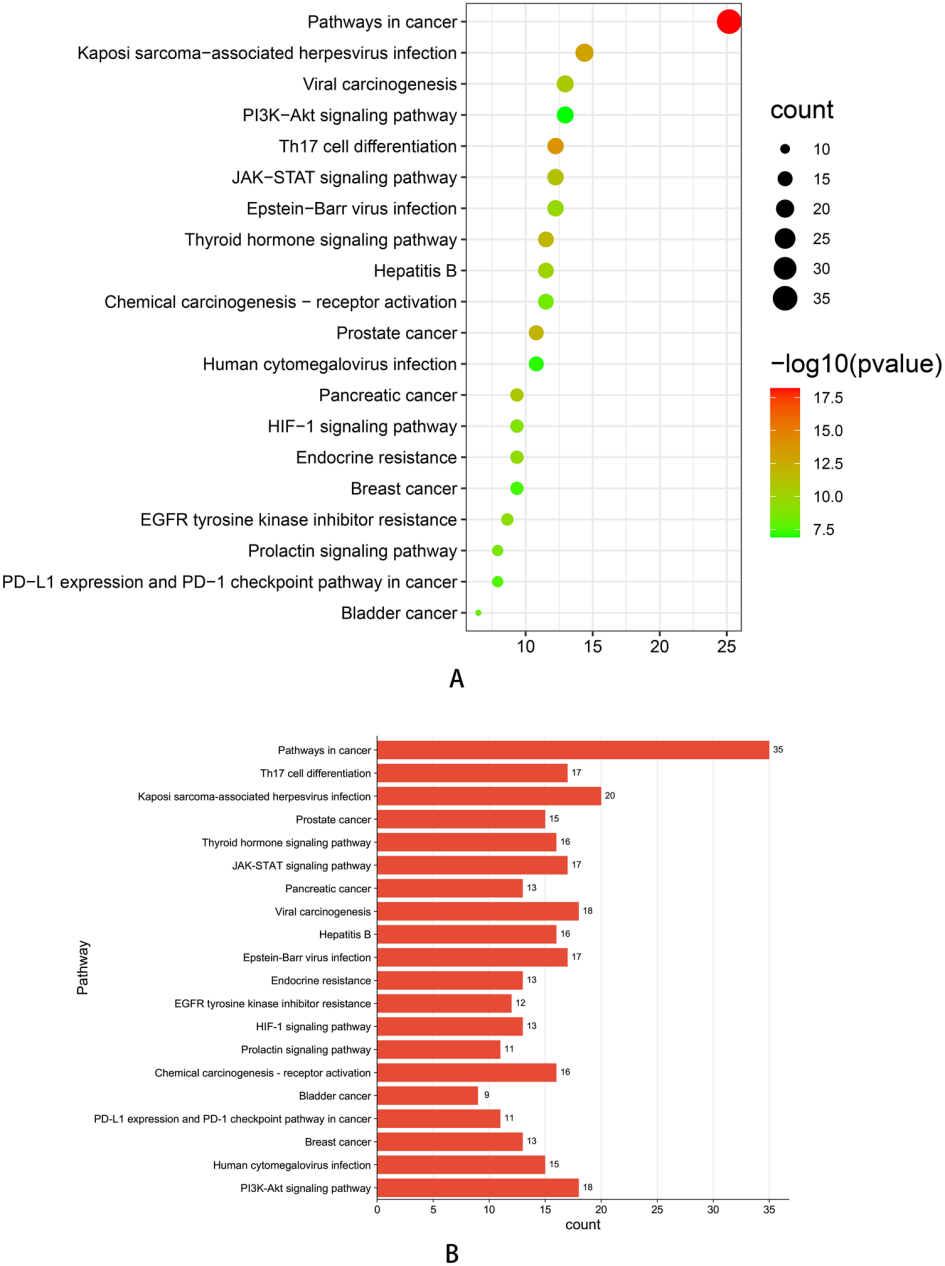
KEGG analysis of STAT3 related genes. KEGG: Kyoto Encyclopedia of Genes and Genomes, (**A**) Enrichment dot bubble diagram (the larger the circular dots, the more genes contained, the darker the color, the smaller *P* value), (**B**) Enrichment histogram (horizontal indicates count. the greater index value, the greater count, the vertical axis indicates pathway).

The top 50 proteins interacting with STAT3 are shown in Supplementary Table S2, and include EP300, CREBBP, EGFR, STAT1, MAPK1, AR (Fig. 7A). After analysis in cytoscape3.9.0 (Fig. 7B), we discovered that there were 19 protein with a greater than the average score (16), such as EP300, CREBBP, EGFR, STAT1, MAPK1, and AR. Among them, EGFR had the largest combined_score (0.931) and NFKB2 had the smallest combined_score (0.502), which was less than the average combined_score (0.521). The top 100 genes interacting with STAT3 are shown in Supplementary Table S3, including PGAP3, GRB7, MIEN1, STARD3, PSMD3, ORMDL3, CDK12, and PPP1R1B. The top five interacting genes were selected for analysis, and the results showed that PGAP3, GRB7, STARD3, and PSMD3 had a significant positive correlations with STAT3 in TCGA cancers (Fig. 8B), such as ovarian serous cystadenocarcinoma (OV) (Fig. 8A). GO enrichment analysis showed that the STAT3-ralated genes and proteins were significantly enriched in the following biological process terms: peptidyl-tyrosine phosphorylation, protein autophosphorylation, transmembrane receptor protein tyrosine kinase signaling pathway, positive regulation of transcription, DNA-templated, and positive regulation of transcription from RNA polymerase II promoter. The cellular component terms included nucleoplasm, cytoplasm, cytosol, macromolecular complex, and nucleus, whereas the molecular function terms included transmembrane receptor protein tyrosine kinase activity, protein tyrosine kinase activity, transcription coactivator binding, non-membrane-spanning protein tyrosine kinase activity, and enzyme binding (Supplementary Table S4). The top 15 biological property terms were screened according to the P-value from smallest to largest (Fig. 9A), and the gene count of each term is shown in Fig. 9B. The top 20 KEGG signaling pathways were also screened according to the P-value, from smallest to largest. The analysis showed that the STAT3-ralated genes and proteins mainly (Supplementary Table S5) acted on pathways in cancer, Th17 cell differentiation, Kaposi sarcoma-associated herpesvirus infection.(Fig. 10A). The gene count for each term is shown in Fig. 10B.

### STAT3 expression is associated with drug sensitivity

**Figure 11:**
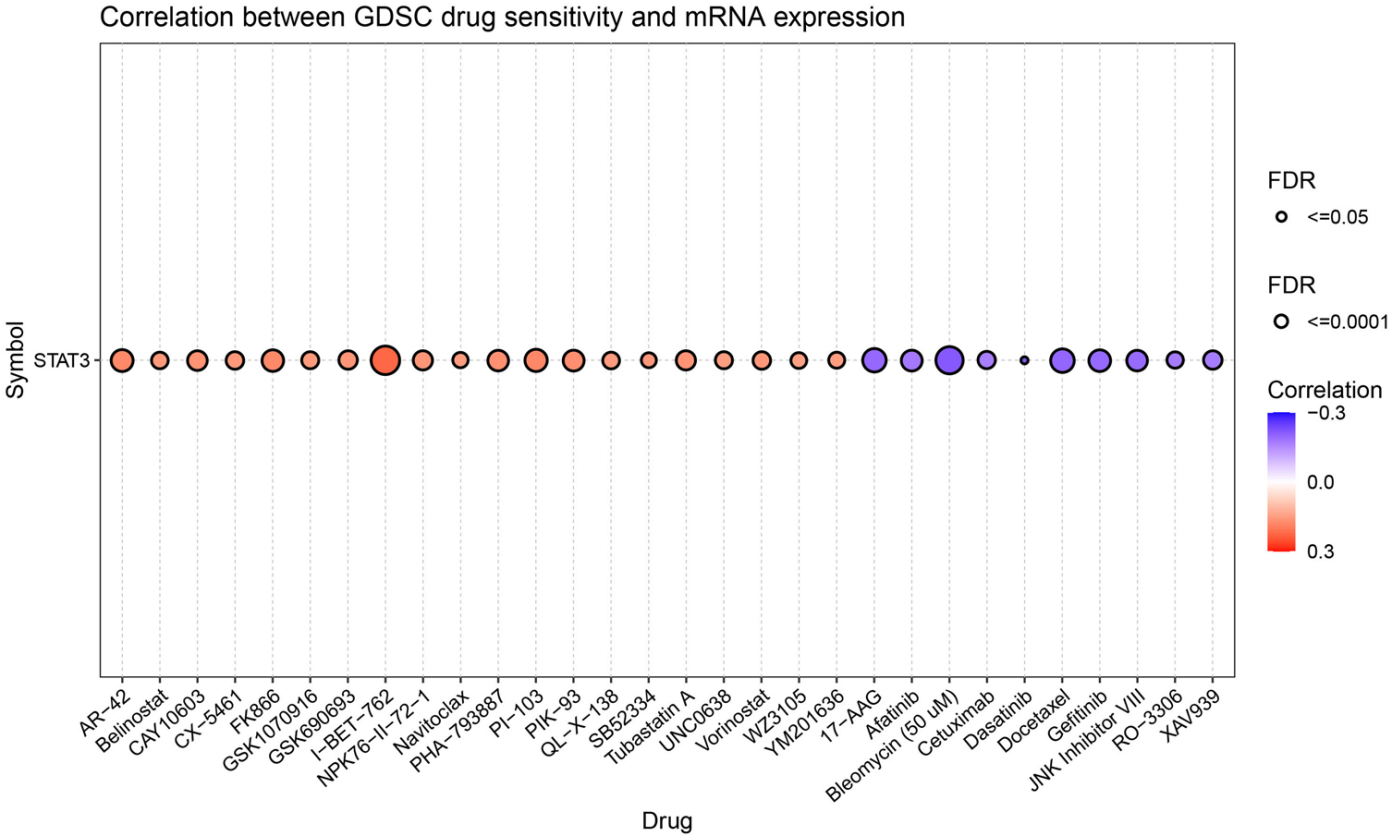
The results of drug sensitivity analysis.

The GSCA database results (Fig. 11) showed a significant negative correlation between STAT3 expression and drug sensitivity (Supplementary Table S6), such as 17-AAG, afatinib, BIRB 0796. A significant positive correlation was found between STAT3 expression and drug sensitivity, including AR-42 and 5-fluorouracil. Among the top 30 drugs, 17-AAG had the lowest FDR, followed by AR-42.

## Discussion

In recent years, cancer has become a prominent public health concern. In recent decades, cancer incidence has grown by nearly 3.9% each year, with a mortality rate of nearly 2.5%^16^. According to the World Health Organization (WHO), cancer was the first or second leading cause of death in 2019^17^. To enhance treatment for patients with cancer and predict prognosis in various cancer patients, immunotherapy and biomarkers have appeared, and have been fruitful in various cancer treatments^18^. Therefore, it is necessary to identify new biomarkers to prevent and treat cancer. Pan-cancer analysis is widely used to identify prospective biomarkers and reliable therapeutic targets^19^. In this study, multiple databases were used in a comprehensive pan-cancer analysis to provide a rationale for STAT3 as a broad-spectrum immunotherapy target in oncology.

Multiple studies have implicated STAT3 in differential tissue expression in multiple cancers. The few studies on STAT3 at the gene level have noted that STAT3 activation is mainly achieved through phosphorylation^20^, and STAT3 has a lower gene copy number, which might be one of the reasons for these findings. It is interesting to note that in the ULACAN database, the levels of STAT3 protein expression were higher than those in normal tissues, showing opposite results to those in Sangerbox. As a geometrical explanation of this phenomenon, research has shown that the levels of mRNA expression do not always correlate with the levels of protein expression, the translation rate of a protein is not identical, and the translational rate determines protein production rates, which are important factors that determine protein production rates^21^. In addition, reports have shown using immunohistochemical analysis that the expression level of STAT3 is significantly higher in patients with glioblastoma multiforme (GBM)^22^, esophageal carcinoma (ESCA)^23^, pancreatic adenocarcinoma (PAAD)^24^ and lung adenocarcinoma (LUAD)^25^ than in controls , which is consistent with our results. In ovarian cancer (OV) and hepatocellular carcinoma (LIHC), STAT3 protein expression was lower than that in normal tissues, but a study has shown that compared with normal tissues, patients with ovarian cancer (OV) had higher STAT3 protein^26^. We speculate that this difference may be due to the small sample size in ULACAN. The GEPIA2 database demonstrated that the expression levels of STAT3 were different among the different breast invasive carcinoma cancer (BRCA* P* = 0.0359) and ovarian serous cystadenocarcinoma (OV *P* = 0.0213) stages. STAT3 expression was found to be closely related to clinical stage (*P* < 0.001), and lymph node metastasis (*P* < 0.001) in breast invasive carcinoma cancer (BRCA)^27^. Studies on ovarian serous cystadenocarcinomas have since confirmed this finding^28^, which consistent with our results. Taken together, these results indicate that the expression of STAT3 differs between normal and cancer tissues in the vast majority of cancers; in particular, STAT3 protein expression is significantly higher in cancer than that in normal tissues.

For OS, the GEIPA2 database showed (Fig. 2) that in the vast majority of cases, patients with kidney chromophobe (KICH, *P* = 0.016; HR = 8.6), brain lower grade glioma (LGG *P* = 0.00096 HR = 1.9), testicular germ cell tumors (TGCT *P* = 0.045 HR = 6e + 08), and uveal melanoma (UVM *P* = 0.038 HR = 2.6) with high levels of STAT3 expression had a significantly worse median survival time than those with low expression. In kidney chromophobe (KICH), the relationship between overall survival of patients and STAT3 expression has not been reported; however, high STAT3 expression has been associated with poor prognosis in patients with Wilms tumors^29^. Similarly, high STAT3 expression promotes glioma cell survival and angiogenesis in brain lower-grade glioma (LGG), which could be another important factor that affects prognosis^30^. Increased STAT3 expression is also relevant in testicular germ cell tumors (TGCT). High STAT3 expression promotes testicular germ cell survival and angiogenesis, which could be another important factor that affect prognosis^31^. STAT3 is highly expressed in uveal melanoma (UVM), which promotes tumorigenesis and cancer progression and plays an important role in tumor prognosis^32^, consistent with our findings. However, in the vast majority of cases, patients with DFS and esophageal carcinoma (ESCA) with high levels of STAT3 expression had a significantly better median survival time than those with low expression. Studies have reported that different expression patterns exist in esophageal adenocarcinoma (ESAD) and esophageal squamous cell carcinoma (ESCA). In esophageal adenocarcinoma, patients with higher STAT3 expression had a superior survival than those with a lower expression^33^. Interestingly, we found that intersections exist in SKCM, which means a potential influence of confounders such as age and gender. In general, the above results showed that STAT3 could be potential biomarkers for multiple cancer diagnoses. However, we drew the conclusion above only from database. Therefore, some confounding factors such as gender, age, and the cancer stages were not taken into account. Hence, these influencing factors should be considered in future studies to validate these findings and draw a more definitive conclusion.

As an oncogene, STAT3 alteration may lead to cancer initiation and progression; therefore, we analyzed STAT3 alterations in different cancers using the cBioPortal database. In diffuse large B-cell lymphoma (DLBC), a study has shown that the mutation of STAT3 induces the occurrence of this cancer^34^. This is consistent with our findings. STAT3 hyperactivation has also been reported in thymomas (THYM)^35^. In addition, the relationship between STAT3 gene alteration and cancer occurrence has also been reported in glioblastoma (GBM) and head and neck squamous cell carcinoma (HNSC)^36^. In summary, alterations in STAT3 are involved in carcinogenesis and cancer progression, especially in hematologic malignancies. Therefore, STAT3 may serve as therapeutic targets.

DNA methylation has been shown to be related to carcinogenesis^37^. Our results showed that the level of STAT3 DNA methylation was increased in the vast majority of cancers. Recent studies have suggested that DNA methylation plays a role in fine-tuning or reinforcing gene silencing^38^, which may be the reason that STAT3 has declined in most cancer types. Few studies have examined STAT3 DNA methylation in cancers. Nevertheless, in pituitary tumors^39^ and leukemia^40^, there have been reports that STAT3 DNA methylation may be early harbinger in cancer, and can promote vascularization^41^. Protein phosphorylation is an important regulatory mechanism that controls protein structure and function, and is thought to play an important role in cancer progression^42^. As an oncogene, STAT3 multiple phosphorylation sites are linked to different cancers. There are reports that STAT3 phosphorylation at Y705 is responsible for activating STAT3 to promote cell proliferation, enhance cell survival and proliferation, promote immune escape, and contribute to lung cancer malignant transformation^43^. Phosphorylation at S727 can activate transcriptional activity^44^, playing an important role in biological activities. The levels of phosphorylated STAT3 have been evaluated as a prognostic biomarker, and the levels of phosphorylated STAT3 at Y705 have been shown to increase the radiosensitization of glioblastoma multiforme cells^45^. In recent years, inhibition of phosphorylation and methylation of STAT3 has been studied as an anti-cancer strategy, indicating that phosphorylation and methylation of STAT3 are promising targets.

The tumor microenvironment has been the subject of intensive investigation in recent years. During tumor initiation and progression, the immune status of the tumor microenvironment is an essential factor affecting tumor progression. Therefore, the tumor microenvironment is considered to be a “the seventh hallmark” of cancer^46^. Our results showed that STAT3 levels were negatively associated with cancer-associated fibroblasts (CAFs) in patients with esophageal carcinoma (ESCA). STAT3 levels have been reported to be inversely correlated with the degree of tumor differentiation in esophageal carcinoma^47^. However, cancer-associated fibroblasts promote tumor growth and progression^48^ which could be one reason for the above results. Similarly, miR-210 transferred by lung cancer cell-derived exosomes acts as a proangiogenic factor in cancer-associated fibroblasts by modulating the JAK2/STAT3 pathway^49^. IL-22 produced by cancer-associated fibroblasts has been reported to promote gastric cancer cell invasion via STAT3 and ERK signaling^50^. In pancreatic adenocarcinoma (PAAD)^51^, STAT3 levels are positively associated with cancer-associated fibroblasts in Pancreatic adenocarcinoma. This is consistent with our findings. The Sangerbox database results showed that STAT3 expression status was significantly correlated with immune infiltration score in 27 cancers, illustrating that STAT3 plays a role in immune responses. Correspondingly, studies have indicated that STAT3 plays a role in the immune responses^52^. This is consistent with our findings. In addition, it has been reported that in glioblastoma, the STAT3 pathway is a key molecular hub in tumor-mediated immunosuppression^53^. STAT3 expression has also been reported to be associated with tumor-associated macrophages differentiation which has also been found^54^. These results show that STAT3 expression is closely related to immune infiltration and may be a novel target for cancer immunotherapy.

To reveal the biological roles of STAT3-related genes and proteins, we performed enrichment analysis. After further visualization, an analysis was performed. STAT3 combined with EGFR showed the highest score. Other investigators have reported similar findings, showing that STAT3 has been linked to EGFR, where EGFR can activate STAT3, thus contributing to cancer progression. For example, EGFR-STAT3 signaling can promote the formation of malignant peripheral nerve sheath tumors^55^, and tumor-derived EGFRvIII can drive STAT3/5 and progression in glioblastoma^56^. In addition, studies have shown that PGAP3, GRB7, STARD3, and PSMD3 are significantly positively correlated with STAT3 in TCGA cancers; however, reports on these are lacking. Based on our results, it has diverse biological functions and participates in diverse signaling pathways, such as Th17 cell differentiation. Th17 cells are immune cells that secrete IL-17 and play important roles in inflammation mediation^57^. Several studies have indicated that STAT3 plays an important role in regulating Th17 differentiation^58^. The JAK-STAT signaling pathway is closely related to the development and progression of various diseases, including autoimmune diseases^59^, autoimmunity^60^ and cancers^61^. We found that it has diverse biological functions and participates in diverse signaling pathways, especially in cancer immunotherapy. Taken together, our data showed that STAT3 is critical in cancer immunotherapy, further illustrating that STAT3 could be a promising target for cancer therapy.

Finally, we analyzed STAT3 function in clinical applications. A significant positive correlation was observed between STAT3 expression and drug sensitivity. AR-42 (previously known as OSU-HDAC42; Arno Therapeutics) has been shown to have potent HDAC-inhibitory activity, which inhibit the GP130/STAT3 pathway to inhibit multiple myeloma cell growth^62^. However, the relationship between STAT3 expression and its drug sensitivity has not been explored. Studies have shown that the Stat3-DNA binding activity and the expressive intensity of phospho-Stat3 protein were lower in 5-fluorouracil drug-resistant cell than in parental cell^63^. Notably, a significant negative correlation was found between STAT3 expression and sensitivity to drugs, such as 17-AAG and afatinib. 17-AAG dampened M1-CM-induced inflammation and catabolism in NPCs by decreasing HSP70 and inhibiting the Janus kinase 2 (JAK2)-signal transducer and activator of transcription 3 (STAT3) pathway^64^. Thus, an increase in STAT3 expression predicted 17-AGG drug resistance. In afatinib-resistant lung cancer cells, inhibition of IL-6R/JAK1 significantly increased the sensitivity to afatinib^65^, and STAT3 expression increased the predicted drug resistance. Collectively, a close relationship exists between aberrant STAT3 expression and drug sensitivity, which may be a cause of drug resistance.

Overall, we found that STAT3 has a good predictive value for cancer prognosis, drug resistance, and immunotherapy. However, even though we systematically investigated pan-cancer information from multiple databases, there were some limitations to this study, such as experimental error and missing data. Additional validation experiments are needed in clinical samples or appropriate models in vitro or in vivo to avoid such bias, and this study provides a rationale for further experimental studies.

## Conclusions

These results indicate that STAT3 can serve as a prognostic, sensitivity prediction biomarker and target of immunotherapy, which is of great value in pan-cancer treatment. However, in-depth studies focusing on STAT3 expression and the tumor immune microenvironment should be conducted to provide a therapeutic strategy based on the immune system.

## Supplementary Information

Supplementary Tables.

## Data Availability

The original contributions presented in the study are included in the article/Supplementary Material; further inquiries can be directed to the corresponding author.
