# Supplementary material for: Comprehensive pan-cancer analysis of STAT3 as a prognostic and immunological biomarker
Source: Sci Rep. 2023 Mar 28;13:5069. doi: 10.1038/s41598-023-31226-2 (PMC10050087; doi:10.1038/s41598-023-31226-2)
Supplement: Supplementary file 1 — Supplementary Tables. [file 41598_2023_31226_MOESM1_ESM.pdf]

Comprehensive pan-cancer analysis of STAT3 as a prognostic and immunological biomarker

Zhibo He[1*], Biao Song[2*], Manling Zhu[2], Jun Liu[1]

Supplementary Table S1 Full names of abbreviations and detailed information of cancer

Cohort	Name
TCGA-ACC	Adrenocortical carcinoma
TCGA-BLCA	Bladder Urothelial Carcinoma
TCGA-BRCA	Breast invasive carcinoma
TCGA-CESC	Cervical squamous cell carcinoma and endocervical adenocarcinoma
TCGA-CHOL	Cholangiocarcinoma
TCGA-COAD	Colon adenocarcinoma
TCGA-COADREAD	Colon adenocarcinoma/Rectum adenocarcinoma Esophageal carcinoma
TCGA-DLBC	Lymphoid Neoplasm Diffuse Large B-cell Lymphoma
TCGA-ESCA	Esophageal carcinoma
TCGA-FPPP	FFPE Pilot Phase II
TCGA-GBM	Glioblastoma multiforme
TCGA-GBMLGG	Glioma
TCGA-HNSC	Head and Neck squamous cell carcinoma
TCGA-KICH	Kidney Chromophobe
TCGA-KIPAN	Pan-kidney cohort (KICH+KIRC+KIRP)
TCGA-KIRC	Kidney renal clear cell carcinoma
TCGA-KIRP	Kidney renal papillary cell carcinoma
TCGA-LAML	Acute Myeloid Leukemia
TCGA-LGG	Brain Lower Grade Glioma
TCGA-LIHC	Liver hepatocellular carcinoma
TCGA-LUAD	Lung adenocarcinoma
TCGA-LUSC	Lung squamous cell carcinoma
TCGA-MESO	Mesothelioma
TCGA-OV	Ovarian serous cystadenocarcinoma
TCGA-PAAD	Pancreatic adenocarcinoma
TCGA-PCPG	Pheochromocytoma and Paraganglioma
TCGA-PRAD	Prostate adenocarcinoma
TCGA-READ	Rectum adenocarcinoma
TCGA-SARC	Sarcoma
TCGA-STAD	Stomach adenocarcinoma
TCGA-SKCM	Skin Cutaneous Melanoma
TCGA-STES	Stomach and Esophageal carcinoma
TCGA-TGCT	Testicular Germ Cell Tumors
TCGA-THCA	Thyroid carcinoma
TCGA-THYM	Thymoma
TCGA-UCEC	Uterine Corpus Endometrial Carcinoma
TCGA-UCS	Uterine Carcinosarcoma
TCGA-UVM	Uveal Melanoma
TARGET-OS	Osteosarcoma
TARGET-ALL	Acute Lymphoblastic Leukemia
TARGET-NB	Neuroblastoma
TARGET-WT	High-Risk Wilms Tumor

Supplementary Table S2 STAT3 The top 50 proteins interact with STAT3

No	Name
1	EP300
2	CREBBP
3	EGFR
4	STAT1
5	AR
6	SRC
7	ESR1
8	MAPK1
9	HDAC1
10	RELA
11	STAT5B
12	HDAC3
13	SP1
14	ERBB2
15	HDAC2
16	CCND1
17	NR3C1
18	HIF1A
19	NFKB1
20	SYK
21	PTK2
22	PGR
23	MYC
24	IGF1R
25	LCK
26	NCOA1
27	MAPK8
28	MTOR
29	JAK1
30	JAK2
31	LYN
32	CDKN1A
33	PDGFRB
34	FOXP3
35	PTPN2
36	PIAS3
37	NFKB2
38	PDGFRA
39	RET
40	HCK

41	IL17A
42	VEGFA
43	BMX
44	JAK3
45	IL17F
46	BLK
47	MAP3K7
48	RPA2
49	GADD45GIP1
50	NDUFA13

Supplementary Table S3 The top 100 gene interact with STAT3

Gene Symbol	Gene ID
PGAP3	ENSG00000161395.12
GRB7	ENSG00000141738.13
MIEN1	ENSG00000141741.11
STARD3	ENSG00000131748.15
PSMD3	ENSG00000108344.14
ORMDL3	ENSG00000172057.9
CDK12	ENSG00000167258.13
PPP1R1B	ENSG00000131771.13
MED1	ENSG00000125686.11
CTB-131K11.1	ENSG00000266469.1
RP11-390P24.1	ENSG00000273576.1
LASP1	ENSG00000002834.17
WIPF2	ENSG00000171475.13
CDC6	ENSG00000094804.9
MRPL45	ENSG00000278845.4
PSMB3	ENSG00000277791.4
SMARCE1	ENSG00000073584.18
FBXL20	ENSG00000108306.11
MED24	ENSG00000008838.17
AC005251.3	ENSG00000219451.3
RPL19	ENSG00000108298.9
EPN3	ENSG00000049283.17
CTB-58E17.5	ENSG00000277182.1
GSDMB	ENSG00000073605.18
AC087491.2	ENSG00000214546.3
PNMT	ENSG00000141744.3
CDH1	ENSG00000039068.18

RP11-458J1.1	ENSG00000278834.1
DLG3	ENSG00000082458.11
MAL2	ENSG00000147676.13
ARHGAP8	ENSG00000241484.9
GGCT	ENSG00000006625.17
GALNT6	ENSG00000139629.15
MSL1	ENSG00000188895.11
LRRC59	ENSG00000108829.9
SPINT1	ENSG00000166145.14
MYO5B	ENSG00000167306.18
RARA	ENSG00000131759.17
CISD3	ENSG00000277972.1
CYP4Z2P	ENSG00000154198.14
SLC2A10	ENSG00000197496.5
ZNF860	ENSG00000197385.5
TMEM62	ENSG00000137842.6
PRR5-ARHGAP8	ENSG00000248405.9
CD2AP	ENSG00000198087.7
TC2N	ENSG00000165929.12
SLC50A1	ENSG00000169241.17
ESRP1	ENSG00000104413.15
MYO6	ENSG00000196586.13
RASEF	ENSG00000165105.9
SMARCD2	ENSG00000108604.15
KRT19	ENSG00000171345.13
RPL23	ENSG00000125691.12
TRAF4	ENSG00000076604.14
CLTC	ENSG00000141367.11
SLC39A11	ENSG00000133195.11
OVOL2	ENSG00000125850.10
CASC3	ENSG00000108349.14
TOX3	ENSG00000103460.16
TMEM86A	ENSG00000151117.8
FAM83H-AS1	ENSG00000203499.10
MARVELD3	ENSG00000140832.9
DDX52	ENSG00000278053.4
MARVELD2	ENSG00000152939.14
DOPEY2	ENSG00000142197.12
PRSS8	ENSG00000052344.15
TMEM241	ENSG00000134490.13
KRT8	ENSG00000170421.11
MANSC1	ENSG00000111261.13
TOM1L1	ENSG00000141198.13
C17orf96	ENSG00000273604.1

HN1L	ENSG00000206053.12
MARK2	ENSG00000072518.20
SERTAD4	ENSG00000082497.11
AP001057.1	ENSG00000232124.1
PTPRK	ENSG00000152894.14
PRR15L	ENSG00000167183.2
KIAA1522	ENSG00000162522.10
CASP6	ENSG00000138794.9
SCYL3	ENSG00000000457.13
KDF1	ENSG00000175707.8
EIF2AK1	ENSG00000086232.12
SRMS	ENSG00000125508.3
GALNT7	ENSG00000109586.11
PRRC1	ENSG00000164244.20
CDS1	ENSG00000163624.5
NME1-NME2	ENSG00000243678.11
C9orf152	ENSG00000188959.9
ALDH3B2	ENSG00000132746.14
DPP3	ENSG00000254986.7
LEO1	ENSG00000166477.12
SPINT2	ENSG00000167642.12
PPAP2C	ENSG00000141934.9
HNRNPF	ENSG00000169813.16
TMEM87B	ENSG00000153214.9
UBE2Z	ENSG00000159202.17
GALNT3	ENSG00000115339.13
ECT2	ENSG00000114346.13
PLEKHF2	ENSG00000175895.3
TRIM26	ENSG00000234127.8

Supplementary Table S4 The result of GO enrichment analyses*

Term	Category	Count	%	PValue	FDR
nucleus	Cellular component	69	49.64029	8.72E-08	4.48E-06
macromolecular complex	Cellular component	21	15.10791	3.26E-08	2.09E-06
cytosol	Cellular component	70	50.35971	9.28E-10	7.95E-08
cytoplasm	Cellular component	71	51.07914	6.84E-10	7.95E-08
positive regulation of transcription from RNA polymerase II promoter	Biological process	31	22.30216	2.02E-10	6.48E-08
positive regulation of transcription, DNA-templated	Biological process	26	18.70504	8.11E-12	3.25E-09
transmembrane receptor protein tyrosine kinase signaling pathway	Biological process	14	10.07194	4.39E-12	2.35E-09
enzyme binding	Molecular function	21	15.10791	4.27E-12	2.55E-10
protein autophosphorylation	Biological process	16	11.51079	1.15E-12	9.25E-10
nucleoplasm	Cellular component	63	45.32374	1.00E-12	2.58E-10

peptidyl-tyrosine phosphorylation	Biological process	15	10.79137	4.63E-13	7.41E-10
non-membrane spanning protein tyrosine kinase activity	Molecular function	12	8.633094	1.57E-14	1.17E-12
transcription coactivator binding	Molecular function	12	8.633094	9.06E-15	9.00E-13
protein tyrosine kinase activity	Molecular function	17	12.23022	9.26E-17	1.38E-14
transmembrane receptor protein tyrosine kinase activity	Molecular function	18	12.94964	5.28E-18	1.57E-15

* Information from www.kegg.jp/kegg/kegg1.html

Supplementary Table S5 The result of KEGG enrichment analyses*

Term	Gene ratio	PValue	Count
Pathways in cancer	25.17986	6.32E-19	35
Th17 cell differentiation	12.23022	1.28E-14	17
Kaposi sarcoma-associated herpesvirus infection	14.38849	7.93E-14	20
Prostate cancer	10.79137	9.74E-13	15
Thyroid hormone signaling pathway	11.51079	1.38E-12	16
JAK-STAT signaling pathway	12.23022	8.11E-12	17
Pancreatic cancer	9.352518	1.54E-11	13
Viral carcinogenesis	12.94964	2.63E-11	18
Hepatitis B	11.51079	9.93E-11	16
Epstein-Barr virus infection	12.23022	2.33E-10	17
Endocrine resistance	9.352518	3.35E-10	13
EGFR tyrosine kinase inhibitor resistance	8.633094	4.69E-10	12
HIF-1 signaling pathway	9.352518	1.18E-09	13
Prolactin signaling pathway	7.913669	2.36E-09	11
Chemical carcinogenesis - receptor activation	11.51079	4.35E-09	16
Bladder cancer	6.47482	7.90E-09	9
PD-L1 expression and PD-1 checkpoint pathway in cancer	7.913669	2.57E-08	11
Breast cancer	9.352518	3.65E-08	13
Human cytomegalovirus infection	10.79137	7.71E-08	15
PI3K-Akt signaling pathway	12.94964	1.21E-07	18

* Information from www.kegg.jp/kegg/kegg1.html

Supplementary Table S6 The result of drug sensitivity analyses

Symbol	Drug	Cor	Fdr	Entrez
STAT3	17-AAG	-0.19573	4.76E-08	6774
STAT3	AR-42	0.179634	2.29E-07	6774
STAT3	Afatinib	-0.17356	5.95E-07	6774
STAT3	5-Fluorouracil	0.130142	0.000256	6774
STAT3	BIRB 0796	-0.1581	0.0005	6774
STAT3	AT-7519	0.121077	0.000583	6774
STAT3	AICAR	0.099945	0.009849	6774
STAT3	BHG712	0.091576	0.011015	6774

STAT3	BAY 61-3606	0.096947	0.013776	6774
STAT3	BIX02189	0.086957	0.016742	6774
STAT3	AKT inhibitor VIII	-0.09801	0.019834	6774
STAT3	A-770041	-0.15473	0.022801	6774
STAT3	AZD8055	0.084079	0.031497	6774
STAT3	AMG-706	-0.10093	0.041551	6774
STAT3	ATRA	0.072344	0.113991	6774
STAT3	AZD6482	-0.06793	0.115103	6774
STAT3	681640	-0.08549	0.126973	6774
STAT3	AS601245	-0.05597	0.285027	6774
STAT3	BEZ235	-0.04968	0.309172	6774
STAT3	AC220	0.04435	0.357112	6774
STAT3	AUY922	-0.04232	0.430348	6774
STAT3	AG-014699	-0.03241	0.475854	6774
STAT3	Axitinib	-0.03478	0.534918	6774
STAT3	AZD7762	0.022313	0.620505	6774
STAT3	A-443654	0.059863	0.716368	6774
STAT3	AS605240	0.016661	0.738758	6774
STAT3	(5Z)-7-Oxozeaenol	-0.01446	0.770341	6774
STAT3	AP-24534	-0.01004	0.841683	6774
STAT3	AZ628	-0.01595	0.865933	6774
STAT3	BI-2536	0.01753	0.897442	6774
